# Public health policy-making for hearing loss: stakeholders’ evaluation of a novel eHealth tool

**DOI:** 10.1186/s12961-020-00637-2

**Published:** 2020-10-29

**Authors:** Giorgos Dritsakis, Lyubov Trenkova, Mariola Śliwińska-Kowalska, Dario Brdarić, Niels Henrik Pontoppidan, Panagiotis Katrakazas, Doris-Eva Bamiou

**Affiliations:** 1 University College London, Ear Institute, London, United Kingdom; 2 Pazardzhik Regional Administration, Pazardzhik, Bulgaria; 3 Department of Audiology and Phoniatrics, Lodz, Poland; 4 Institute of Public Health for the Osijek-Baranja County, Osijek, Croatia; 5 University of Osijek, Faculty of Dental Medicine and Health, Osijek, Croatia; 6 Eriksholm Research Centre, Oticon A/S, Snekkersten, Denmark; 7 Biomedical Engineering Laboratory, Institute of Communication and Computer Systems, Athens, Greece; 8 Biomedical Research Centre, National Institute for Health Research, London, United Kingdom

**Keywords:** hearing loss, public health, policy-making, EVOTION platform, big data, SWOT

## Abstract

**Background:**

Hearing loss (HL) affects 466 million people of all ages worldwide, with a rapidly increasing prevalence, and therefore requires appropriate public health policies. Multi-disciplinary approaches that make use of eHealth services can build the evidence to influence public policy. The European Union-funded project EVOTION developed a platform that is fed with real-time data from hearing aids, a smartphone, and additional clinical data and makes public health policy recommendations based on hypothetical public health policy-making models, a big data engine and decision support system. The present study aimed to evaluate this platform as a new tool to support policy-making for HL.

**Methods:**

A total of 23 key stakeholders in the United Kingdom, Croatia, Bulgaria and Poland evaluated the platform according to the Strengths, Weaknesses, Opportunities and Threats methodology.

**Results:**

There was consensus that the platform, with its advanced technology as well as the amount and variety of data that it can collect, has huge potential to inform commissioning decisions, public health regulations and affect healthcare as a whole. To achieve this, several limitations and external risks need to be addressed and mitigated. Differences between countries highlighted that the EVOTION tool should be used and managed according to local constraints to maximise success.

**Conclusion:**

Overall, the EVOTION platform can equip HL policy-makers with a novel data-driven tool that can support public health policy-making for HL in the future.

## Introduction

### Hearing loss as a public health issue

Hearing loss (HL) affects approximately 466 million people worldwide, i.e. 6.1% of the world’s population, with the total number of people with HL estimated to rise to over 900 million by 2050 [1]. It is the fifth leading cause of years lived with disability, higher than diabetes and visual impairment [2]. It has been associated with a higher risk of dementia, mental illness, and depression and with an adverse overall effect on general health [3]. It also has important economic consequences, including work discrimination, reduced productivity, unemployment and early retirement and, as a result, loss of income [4, 5]. The most common causes of HL are ageing, noise exposure, complications at birth, genetic causes, infectious diseases, chronic ear infections or ototoxicity. Some types of HL are preventable, and thus amenable to public health (PH) policy-making and legislation. For example, 60% of childhood HL is due to preventable causes, where 1.1 billion young people are at risk from exposure to loud sounds on personal audio devices and in noisy entertainment venues [6]. People with HL can benefit from early identification and from the use of assistive devices such as hearing aids (HAs) or cochlear implants, captioning, and other forms of educational or social support. However, HA users still face significant challenges such as listening in noisy environments, poor sound quality and difficulties in selecting among predefined settings. As a result, many adults, especially elderly, do not accustom to their HAs and do not use them, with huge cost implications for health systems [7]. It has been reported that up to 40% of people fitted with a HA either fail to use it or do not benefit from it [8]. In the United Kingdom, the annual cost of unsuccessful HA use has been estimated to be over £40 million of NHS funding [9]. Moreover, it is unclear whether HAs are equally effective for adults with different degrees of severity of HL [10]. Interdisciplinary, holistic approaches for the management of HL can yield valuable insights into HA uptake, use and outcomes, so that changes can be made at the population and PH policy-making level [11]. It is crucial that countries develop long-term evidence-based PH programmes and policies to raise awareness for HL and for HL prevention, early diagnosis and effective interventions in order to support healthcare systems and to ultimately promote the social inclusion of the hearing-impaired population.

### Policy-making challenges and eHealth

In the last decade, in health policy-making there has been a transition from traditional methods (e.g. use of expert opinion or data from single randomised controlled trials) towards more evidence-based approaches at the population level. Such techniques currently used are systematic reviews, national routine data monitoring and expert groups [12–15]. Even though these techniques overcome previous limitations, their validity and reliability is not always clear and data are likely to be out of date and therefore not sufficient for clinical or PH action [16]. Collaborative approaches as a whole pose additional challenges as PH policy-makers may not be able to engage effectively with clinicians, healthcare professionals or other experts who could support the decision-making process, for example, due to the use of different terminology [17].

Currently, there is an increasing interest in and use of eHealth services, such as databases, big data analytics or mobile applications, to help healthcare professionals communicate better, enable patients to access the care they need quickly and easily, and strengthen health systems worldwide [18]. The use of eHealth can also benefit public policy-making by improving planning and resource allocation, cost-efficiency, health service delivery, real-time monitoring, personalised services and preventive measures [19]. A number of policy modelling and formation techniques, processes and guidelines already exists such as ontology models or assessment tools for health services, technologies and impact (e.g. [20–22]). However, to our knowledge, no online policy-making tools supporting the specification of policies or policy-making processes are available to date. In order to make use of the tremendous potential of eHealth for policy, we need to overcome several challenges with regards to awareness, quality of the tools, access and costs. For developing countries in particular, cultural and educational issues have been reported as huge obstacles towards the use of eHealth applications [23]. On the other hand, well established healthcare systems may have the ability to integrate eHealth services more easily [24]. The field of audiology, in particular, can massively benefit by the use of new technologies such as smart HAs and mobile applications linked to them, HA data logging or population databases [25, 26]. However, we are not aware of any tools that incorporate data analysis capabilities and PH decision support for HL. With the rapid increase in the number of people affected by HL and in view of several gaps in current knowledge in the field (as sated above), building dynamic data-driven systems that are linked to hearing devices, existing clinical databases and other tools may generate evidence to not only improve hearing care but also to inform decisions at the population level.

### The EVOTION approach

**Fig. 1:**
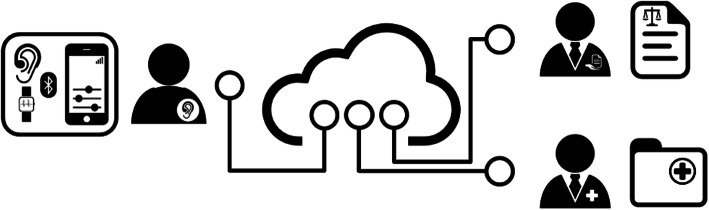
Schematic representation of the EVOTION platform. Middle: the EVOTION Data Repository collecting data from various sources. Left: Hearing aid users transmitting data via smart hearing aids, smartphones and potentially via smartwatches if available using a Bluetooth connection. Bottom right: healthcare professional entering data from the clinic either directly through a dashboard of the platform or through existing clinical databases connected to the EVOTION Data Repository. Top right: policy-maker running queries on the EVOTION database through the dashboard in order to answer specific public health issues. Source: Pontoppidan 2019 [27]. Used with permission

The collaborative European Union-funded EVOTION project has built an integrated platform to support the formulation of PH policies related to HL prevention, diagnosis and rehabilitation with the use of big data [28, 29]. EVOTION’s overarching ambition is to promote evidence-informed policy-making in hearing healthcare, in line with the increasing use of eHealth applications worldwide. The EVOTION platform enables, among others, (1) static and real-time data from HA users to be collected from various sources and continuously fed into a data repository and (2) the application of big data analytics (BDA) techniques via a BDA engine and based on pre-defined public health policy decision-making (PHPDM) models. A PHPDM model is a mathematical structure that processes two or more sources of evidence and projects out the health outcomes associated with alternative policies [30]. The EVOTION PHPDM models do not define PH policies as such, but rather the statistical analyses and the data required to support policy decisions and, based on these, plausible decisions for different scenarios within the context of the EVOTION project [31]. Subsequently, the results of the BDA tasks can yield policy recommendations that can then be used by relevant stakeholders through a decision support system. The EVOTION decision support system enhances and refines the PHPDM models [32]. To enable the validation of the EVOTION platform, a multi-centre clinical study with 1080 HA users has collected big data, including data from smart HAs and a mobile application as well as audiological, demographic, cognitive and quality of life data [33]. All data were hosted in the EVOTION Data Repository (EDR). The total amount of data at the end of the project was 60,939,630 data points. Analytics are performed through a dashboard of the platform where the end-user can select the type of analysis they want to run and then the EVOTION data on which they want to run the analysis. Figure 1 is a schematic view of the architecture of the EVOTION platform, with the key components explained.

### Aim

The aim of the present study was to evaluate the EVOTION platform as a novel innovative tool to support PH policy-making in the domain of HL from the point of view of key stakeholders, who are potential future end-users of the platform.

## Methods

In order to demonstrate the functionalities of the EVOTION platform to relevant stakeholders and collect evaluation feedback, a series of workshops were conducted between April and September 2019 by four different project partners in their home countries, as follows:

- London, United Kingdom, organised by University College London
- Osijek, Croatia, organised by the Institute of Public Health for the Osijek-Baranya County
- Sofia, Bulgaria, organised by Pazardzhik Regional Administration
- Warsaw, Poland, organised by Nofer Institute of Occupational Medicine

Workshops were hosted by these four specific organisations because they were the partners of the EVOTION project responsible for delivering the PH policy evaluation of the platform and had the necessary expertise and resources. The choice of these countries enabled the evaluation of the platform in health systems and populations with different demographic and economic characteristics, budgets, PH priorities and other constrains with regards to public policy decisions. The following section describes current PH policy-making local methods in each country.

### The workshops

All four workshops included the following key components:

1. Presentation 1: Overview of the project and policy-making evaluation
2. Presentation 2: Insights from preliminary data analysis
3. Demonstration of the EVOTION platform
4. Focus group discussion

**Fig. 2:**
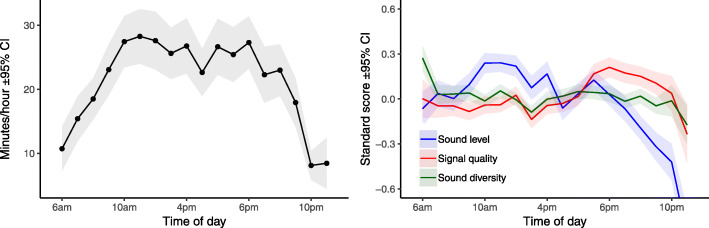
Preliminary results from EVOTION dynamic data analysis. Average hearing aid usage over time (left) and how the sound level, sound diversity and signal quality, i.e. signal-to-noise ratio, describe the acoustic environment (right). Source: Christensen et al. [34]. Used with permission

Presentation 1 provided background to the EVOTION project and its aims, explained the clinical study and described the different components of the platform as well as the types of data that were collected [33]. Presentation 2 focused on preliminary results from analysing a sub-sample of the EVOTION dynamic data, i.e. data describing the use of HAs in different acoustic environments by different HA users. We demonstrated the analytical methods we had so far applied to the data and how these methods are being implemented in the EVOTION BDA engine. More specifically, we presented how much HAs were used over time by the participants of the EVOTION clinical study and the acoustic environmental context (i.e. how sound characteristics varied) over the same time period; this is illustrated in Fig. 2. Our findings showed a positive correlation between HA usage and overall sound level and diversity and a negative correlation between HA usage and overall signal-to-noise ratio [34]. We also presented preliminary findings suggesting how the EVOTION HA data can be used to predict temporary threshold shifts and noise-induced hearing loss for individuals and the general public [35, 36].

**Fig. 3:**
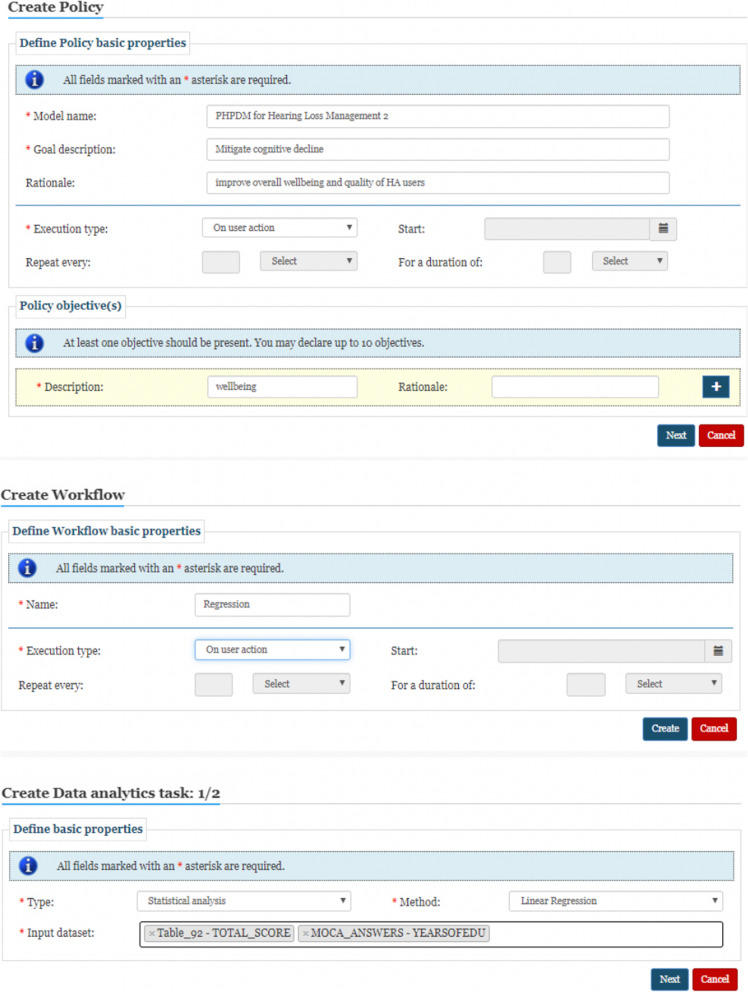
Snapshots of the EVOTION dashboard. Top: creation of a policy using pre-defined Public Health Policy Decision-Making models. Middle: creation of a workflow within that policy specifying the statistical techniques to be used. Bottom: Creation of a data analytics task within the workflow by specifying the types of data from the EVOTION Data Repository to be used. Source: Basdekis et al. [37]. Used with permission

The EVOTION platform demonstration (item 3) included the user interface of the dashboard, how to perform queries in the EDR, use of analytic tools (including the creation of tasks, workflows and policies) and results visualisation [38]. The demonstration focused on how the end-user can run analytics in three steps – (1) the creation of a policy specifying which PHPDM model it should be linked to, (2) the creation of a workflow within that policy specifying the statistical techniques to be used and (3) the creation of a data analytics task within the workflow by specifying the types of data from the EDR to be used (Fig. 3). This demonstration enabled stakeholders to see the actual process and the functionalities of the platform. It should be noted that, at the time of the workshops, these components had not been finalised and therefore participants were not able to see the whole set of functionalities or get any hands-on experience with the tool. The focus group discussion part of the workshops was facilitated by a set of questions used across all workshops and is detailed below.

### Participants

Purposive sampling was employed by each of the 4 institutions to select professionals that would be potentially involved in making, executing, recommending or discussing policy decision in the area of HL such as PH, policy or audiology experts. In total, 23 professionals participated in the evaluation across the 4 countries with 5 to 7 attendees per workshop. Participants represented a wide range of expertise. In the workshop in London, they were mainly high-profile national HL experts with advisory roles in policy-making (*n* = 5). In Croatia, they were experts in implementing regional-level PH policies (*n* = 7). The workshop in Bulgaria was attended by national-level general (but not HL) PH policy experts advising on PH policies, who were a representative sample of experts from all key quadruple-helix stakeholders of the policy-making process (*n* = 6). In Poland, attendees were again national and governmental PH experts involved in drafting PH policy legislation/regulations focused on noise exposure and HA distribution and financing (*n* = 5) (Additional file 1). Engaging such a range of stakeholders ensured that the assessment of the platform was based on different perspectives depending on the expertise of each of the partners and institutions hosting the workshops. Keeping the workshops relatively small ensured an in-depth discussion and gave all attendees the opportunity to provide detailed feedback.

### Focus group discussion

Following the EVOTION platform demonstration (step 3 above), we conducted a semi-structured focus group discussion to explore the stakeholders’ views on the platform following the Strengths, Weaknesses, Opportunities and Threats (SWOT) approach. SWOT is a method originally developed to systematically analyse an organisation’s strategic position in relation to its competitors or for the purposes of project planning [39]. It consists of identifying external opportunities and threats as well as the internal strengths and weaknesses of a company or a project. Based on the interaction between these factors, strategic plans can be developed. SWOT has grown in popularity and has been used in multiple fields, including the healthcare sector [40, 41]. The SWOT methodology was chosen because the EVOTION platform was developed as a novel tool in the market and, even though there is no other tool to compare it directly with, it was necessary for the tool to be assessed for its advantages and disadvantages in the context of health policy-making as a whole.

In order to prompt discussion, the focus group facilitator in each country asked questions such as:

- Please tell us about the strengths or advantages of the EVOTION tool. What do you think is better or unique compared to other similar tools?
- Please tell us about the weaknesses of the EVOTION tool. What do you think could be improved or avoided?
- Please tell us about the opportunities that the EVOTION tool creates.
- What may be the obstacles in the use of EVOTION as a PH policy-making tool for HL in the future?
- Would you be willing to use it yourself or recommend it to others in the future if it was finalised and available to use?

The last question was not strictly related to the SWOT methodology but was key to the aim of the study. The discussions were audio-recorded and transcribed. Based on each recording, a country-specific SWOT was drafted and, based on these four SWOTs, common SWOT themes were identified.

### Workshop discussion data analysis

The focus group data was analysed thematically according to the SWOT methodology. Specifically, in the context of the present study, the following definitions were used:

- Strengths: internal features of the EVOTION platform that give it an advantage over other similar tools in the field.
- Weaknesses: internal limitations or disadvantages of the tools over other similar ones.
- Opportunities: characteristics of the tool that give it a future potential in relation to the broader environment. This is in contrast to the traditional definition as elements of the external environment that an organisation or project could exploit for their own benefit.
- Threats: the interaction between the platform and external factors that could pose a risk to the tool reaching its full potential, in contrast to the traditional definition of elements of the environment external to the platform that can cause problems.

The country-specific SWOTs were created by two members (at least one of which had participated in the workshop) of each of the four research teams. Initially, themes were identified in the data of each of the four workshops within each of the above SWOT domains by one member of each team, broadly following the steps by Braun and Clark [42] and using as a priori domains the four SWOT categories. For each SWOT, themes were then reviewed by the second member of the team, discussed and final SWOTs were developed. The themes from the four SWOTs were then compared to each other for similarities and differences and an overall framework with common themes was produced (see below) as well as themes brought up to a single country only (see below). This process involved merging, splitting, renaming themes and moving them across domains. The process was led by the first author but involved discussion with all co-authors. Comments regarding the potential future use of the tool were also extracted separately from the SWOTs from each research team, summarised by the first author and are presented in section 3.3.

## Results

### Common SWOT themes

**Fig. 4:**
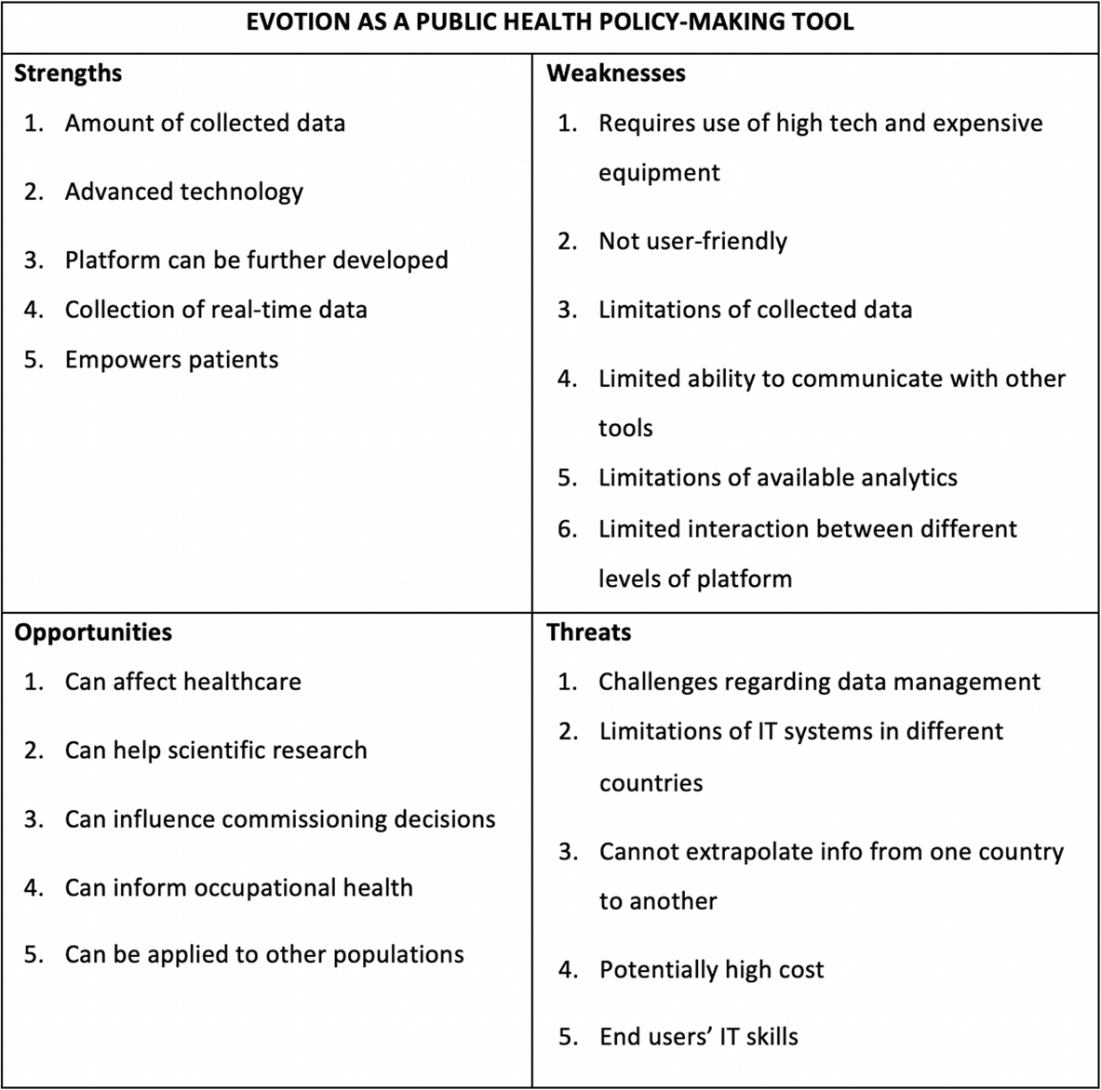
EVOTION evaluation results. Summary of common themes from all four workshops produced after identifying themes in the SWOT analysis of each of the workshops and then comparing themes from the four countries to each other

The SWOTs of the EVOTION platform that were identified in more than one of the workshops are summarized in Fig. 4 and explained here in detail with examples. The letters in the brackets indicate the countries in whose SWOT each theme was identified, i.e. UK for the United Kingdom, C for Croatia, B for Bulgaria and P for Poland.

#### Strengths

1. Size of database and amount of collected data [UK, P, B]
2. Technologically advanced solution and high level of technical integration [C, B]
3. The platform has potential to be developed (e.g. by simplifying/automating processes, collecting data from other sources) [UK, B, P]
  “*Potential to embed more analyses*.” (United Kingdom)
4. Collection of data from sources where measurements are difficult, e.g. real-time data [UK, P, B]
  “*Capability to obtain statistics concerning the effect of staying in noise-exposed environment for users of HAs. Thus far, these statistics were not as easily accessible*.” (Poland)
5. Empowers patients by offering increased capabilities, e.g. HA controls [UK, P, C, B]
  “ *…giving increased capabilities as compared to hitherto used devices, including the capability to customize hearing aids*.” (Poland)

#### Weaknesses

1. The platform requires high tech and expensive technology and equipment, e.g. smart HAs [UK, P]
  “*Requires advanced technologies, thus might be rejected by individuals not accustomed to novel technical and informational solutions, particularly elderly people.*” (Poland)
2. Not user-friendly, complicated mechanism [UK, P, B]
  “*Currently the mechanism of data collection and analysis is overcomplicated. The operational model is difficult to explain and is not fully understood by representatives of governmental and non-governmental entities which do not come in direct contact with hearing-impaired individuals.*” (Poland)
3. Limitations of collected data, e.g. more detail needed regarding the listening/acoustic environments, noise exposure or qualitative/significant other data [UK, P, B]
  “*We need qualitative and significant others’ information … We need more detail re specific listening environments*.” (United Kingdom)
4. Limited ability to engage, communicate or be merged with other tools or databases [UK, P, C]
5. Limitation of available analytics, e.g. tool not fully functional at the time of workshop, lacks major analytic capabilities required for PH policy decision-making [C, P, B]
  “*Not enough emphasis on PH policy-making processes, e.g. no economic evaluation included*.” (Bulgaria)
6. Limited interaction between different levels of platform, e.g. limited access to database [UK, P]

#### Opportunities

1. Potential to affect healthcare [UK, P]
  “*Faster response to problem … can provide a quick response to HL problems as they arise*.” (United Kingdom)
2. Data can be used for scientific research [UK, C]
  “*Can show how HA compliance translates to real life benefit. Compared to other health interventions hearing care has better compliance, convincing evidence of 60–80%. How does that translate in real world benefit, how does it impact on rest of healthcare, does it save money?*” (United Kingdom)
3. Can influence commissioning decisions, e.g. facilitate control over state budget’s expenditures on HA refunds by better fitting [UK, C, P]
  “[Could use the platform to] *lower end costs in healthcare … better integration, reduction of treatment costs resulting from bad hearing care regulation*.” (Croatia)
4. Potential to inform occupational health regulations and law in the future, e.g. limits on permissible noise volume in personal electronic devices or implementation of Temporary Threshold Shift model in new generation HAs [UK, C, P, B]
  “ *… could take into account prediction of noise exposure in changing occupational health requirements*.” (Bulgaria)
5. Can potentially be applied to other populations or specialties, given that various sources of PH data are more and more readily available [UK, B, C, P]
  “*Inclusion of children in platform usage … user-friendly app in the form of a game?*” (Croatia)
  “*Opportunity to apply EVOTION technology and PH data collection methods to other areas beyond HL*.” (Bulgaria)

#### Threats

1. Challenges regarding data management, retention and security, e.g. compliance with data protection regulations or hacking risk [UK, C, B, P]
  “*Security risks, e.g. issue with Bluetooth connection between HA and mobile phone*” (Bulgaria)
2. Hard to implement due to limitations of IT systems in different countries [C, P, B]
  “*Unsatisfactory quality of data in healthcare IT system … Bad inter-sector and healthcare IT connection in different institutions in healthcare*.” (Croatia)
3. Cannot extrapolate information from one country to another due to differences between countries [UK, B]
  “*Not possible to validate the statistical representativeness of the cohorts on a national scale* [as the] *clinical study* [was] *carried out in several countries.*” (Bulgaria)
4. Potentially high cost due to complexity, etc., e.g. challenge of financing of platform due to tight public budgets [UK, P, C]
  “*Ensuring continuous financing of* [the] *EVOTION platform implementation* [is a weakness] *… Limited national health insurance funds*.” (Croatia)
5. Range of economic power, IT literacy or knowledge, or analytic skills of patients and potential end users, e.g. elderly people [C, P, B]
  “*Unequal availability for users regarding economic power and skills … especially the elderly*.” (Croatia)
  “*Insufficient technical infrastructure and capabilities at the end user side could limit the applicability and exploitation of the system’s functionalities to its full potential. Insufficient knowledge of end users about analytics, neural networks, etc. A constraint for the applicability and exploitation of the platform*.” (Bulgaria)

### Country-specific SWOT themes

In addition to the above common themes, certain themes were specific to one country only. This was possibly related to the different expertise of the stakeholders who participated in each of the workshops.

In the United Kingdom, participants raised the potential to identify problems with the data collection process itself as an opportunity but also highlighted that the fact that the population the data were collected from was skewed and difficulties to use the tool towards HL prevention as weaknesses.

“*Potential at data acquisition level…redefining goals, considering why you collect these specific data*.” (United Kingdom)

In Croatia, the development of the platform was seen as an opportunity that brought together experts from different institutions and disciplines and trained them with advanced IT tools.

“*Existence of adequate experts for work on the platform.*” (Croatia)

In Bulgaria, professionals brought up the opportunity to use commonly adopted user-experience conventions to help improve the user experience with the platform but also recognised that EVOTION is a very specialised tool in a very small area of the market.

“*The tool could be considered as intended for a very tight market segment of the PH area whereas other PH areas, e.g. control and policies in the areas of coronary diseases, or treatment and long-term care for diabetics are considered of higher priority in national healthcare – upgrading the current tool for such areas could necessitate a significant modification for data collection*.” (Bulgaria)

Finally, Polish policy-makers acknowledged that other tools currently being developed by HA manufacturers may surpass EVOTION but also thought that the platform can benefit people with normal hearing as well as the hearing impaired through prevention programmes.

“*The product might also be beneficial for persons with correct hearing, through prevention programs*.” (Poland)

### Future use of the EVOTION tool

In addition to the SWOT analysis above, professionals commented on whether they would be willing to use the EVOTION tool in the future and how. All stakeholders were generally very positive about using the platform in the future and gave examples but also mentioned certain obstacles or prerequisites.

Specifically, feedback from Poland involved the potential use of the platform (1) as part of campaigns and prevention programmes for HL, (2) to develop guidelines for employers or people with HL with regards to noise protection at work, or (3) at a higher level use by ministries for specifying the criteria for the provision and refund of HAs.

“*Use by the Ministry of Health for specifying regulations on required conditions for selecting and refund criteria of hearing aids…for specifying regulations on hiring persons with hearing loss using hearing aids, for positions exposed to noise*.” (Poland)

Along the same lines, data from Bulgaria involved the potential adaptation of the tool to monitor the occupational environment for noise control but also in integration with other eHealth data repositories or by patients’ organisations to collect feedback. On the other hand, United Kingdom stakeholders stressed the need to clearly define the intended audience and use of the EVOTION platform. Use by governments or charities was mentioned only as one way to change policy.

“*Need to define which policies we are trying to influence*.” (United Kingdom)

Further to this, Bulgarian participants suggested that a number of functionalities should be added to enhance the tool, including the assessment of the impact of the recommended policy decisions especially with economic measures (cost-benefit, cost-effectiveness, incremental cost analysis) and short- and long-term monitoring and evaluation of these policies overall. They also suggested that analyses of budgetary implications and the consistency of the recommended policies with existing ones in the same area may add to improvements of the tool.

## Discussion

### Summary of findings

The present study collected feedback from a range of professionals involved in PH policy-making in four different countries on the SWOTs of the EVOTION platform as a tool that can support policy-making for HL. Participants were generally very enthusiastic about the capabilities and potential of the tool. Among its advantages were the amount of collected data (Fig. 4, Strength 1), the high-end technology implemented (Strength 2) and the collection of real-time data (Strength 4). There was consensus that the platform can use such capabilities to influence commissioning decisions (Opportunity 3), inform occupational health regulations and laws in the future (Opportunity 4), and benefit healthcare as a whole (Opportunity 1). For example, as highlighted by a participant of the London workshop, in the context of HL treatment in particular, the tool could be used to show how HA compliance translates to real-world benefit, how it impacts on the rest of healthcare and if it is cost-efficient. Given the lack of other available eHealth tools, these strengths and future potential make the EVOTION platform a promising new entry in the market that could generate data to inform the field of HL but also PH policy decisions at a higher level.

On the other hand, stakeholders highlighted a number of limitations of the tool itself such as the high requirements for the end-user (Weakness 2) or the difficulty in accessing various levels of the platform (Weakness 6). Risks that need to be considered for the successful future use of the tool were also identified, including challenges regarding data management (Threat 1), the potential high maintenance cost (Threat 4), and difficulties in across country use due to differences in IT systems (Threat 2) or the IT skills of the population (Threat 5). These limitations and risks stress the fact that there is still work to be done for the EVOTION tool to be properly exploited and to reach its full potential. This is not surprising for an innovation incorporating a number of different components and implementing such advanced technology. Some of the weaknesses and threats were addressed in the finalisation of the platform after the workshops (e.g. additional analytics) or are ongoing work (e.g. data management and maintenance). Other considerations are related to the different needs, health systems mechanisms and resources available in different European Union countries and would need to be addressed in a country-specific and setting-specific (e.g. national versus regional) manner for the successful implementation of the platform and adoption in the future. However, the platform’s flexibility to change and further develop in response to evolving stakeholder needs in the future was demonstrated to the stakeholders and appreciated by them (Strength 3). For example, after the workshops, a new simulation functionality was added that allows the enhancement of the PHPDM models depending on user requirements and, in this way, it complements the BDA results and supports even further the policy recommendation process [31].

### Country-specific considerations

The EVOTION evaluation workshops included stakeholders with a wide range of expertise who represented countries with different socioeconomic characteristics and PH systems in terms of resources, organisation and approach. Certain themes identified in the SWOT analyses reflect different PH resources available across the four countries, such as the “*limited ability of the platform to engage with other tools*” brought up mainly in the London workshop (Weakness 4). In the United Kingdom, there are other NHS databases that the EVOTION platform may need to communicate with, while in Bulgaria, for instance, this may be less of an issue as the platform may provide a health database for HL or the use of HAs where none currently exists.

If we consider the results of the SWOT analysis in the context of the current PH policy-making system of each country, we can see an additional advantage of the use of platforms like EVOTION, that of raising awareness within the policy-makers as well as shaping policy for the benefit of the population. For instance, the majority of the Croatian stakeholders who participated in the workshops reported a lower level of HL awareness compared to the United Kingdom or Poland, which have more organised HL services. In this case, use of a system like EVOTION would help increase awareness among professionals for HL in particular. Additionally, Croatian stakeholders reported as strengths of the EVOTION project overall the fact that it trained experts from different institutions to work with specific IT tools and this way it generated ideas for the establishment of new departments for international collaboration at different institutions in the future. This is even more important given that it has been previously reported that PH stakeholders have difficulty liaising with other relevant stakeholders such as non-governmental organisations and regional authorities and that they may not be appropriately equipped and familiar with the use of new technologies and services for policy-making in the area of health [17].

### Future use of the EVOTION tool

Generally, PH policy-making stakeholders at a national and local level who participated in the present study are open to welcoming such a technological approach to their traditional activities but indicated that either they were not the ones who would be using the tool or that a number of issues need to be addressed until they can actually use it in decision-making. The Croatian stakeholders stressed the uniqueness of the tool in the area of policy-making and participants in Poland and Bulgaria mentioned specific examples of how the tool could be used and for what purpose. Moreover, some of them, as explicitly reported in Bulgaria, already have knowledge of neural networks and big data technologies or are willing to learn, which is an advantage given the complexity of the tool. In addition, specific campaigns and awareness-raising approaches will have to be adopted for stakeholders at the higher level of decision-making to recognise the advantages and, hence, be willing to dedicate public funding and issue guidelines for using such platforms as part of the routine policy-making practice. However, United Kingdom stakeholders did report that they themselves were not in a position to use it and that the target future users of the tool and the ways in which it can shape policy should be specified. This comment may reflect the different ways policy-making is made across countries and the different roles of stakeholders across workshops. For instance, in the United Kingdom, health policy-making is done at a high governmental level and none of the United Kingdom workshop participants held such an executive position to use the EVOTION tool for actual decision-making. The potential future costs, for Croatia and Bulgaria in particular, were also reported to be a crucial factor both for purchasing the platform and for the assessment of potential policy decisions offered by the platform. Certain improvements could help the uptake of the tool by potential users. For instance, integrating health economics analytics in future versions would be important in order to fully meet the needs of PH policy stakeholders. Additionally, the provision of a free trial or an open-source version could give future end-users the chance to test the tool and facilitate take-up. In order to assist future users, a comprehensive guide is also being prepared and will be made publicly available for future reference and use by any interested potential user [43].

### Evaluation methodology

The present study obtained feedback from a range of potential future users of the EVOTION tool based in three European regions with distinctly different constraints with regards to PH decision-making (Western Europe, Balkan region, Eastern Europe). The stakeholders seemed to understand and appreciate the various components and capabilities of the platform. Their feedback has valuable implications for the improvement and exploitation of the EVOTION technologies towards evidence-based policy-making for HL. A major limitation of the evaluation process used in the present study was the fact that, at the time of the workshops, the platform was not complete and did not allow stakeholders to have hands-on experience with the tool. This was due to practical reasons, related to the timeframe of the delivery of the EVOTION project as a whole. We also acknowledge that this evaluation represents a very small number of professionals from only four countries selected on the basis of their participation in the EVOTION consortium and it is unclear if the results are generalisable.

## Conclusions

The EVOTION platform is an innovative eHealth tool developed at an opportune moment for national and international strategies to harness technologies to improve healthcare delivery and PH policies. Key stakeholders from different PH settings in four European countries agreed that the implementation of the tool would lead to substantial benefits for the formulation of PH decisions for HL by providing highly relevant and extensive evidence and by facilitating timely decisions in response to emerging situations and needs in the future. However, a number of improvements need to be made in the platform, risks to be carefully considered, and the intended use and target audience need to be specified. As long as we can overcome these obstacles, stakeholders expressed an interest to utilise the EVOTION tool in their respective role and field of work to generate evidence-based, high-quality policy recommendations.

## Supplementary information

**Additional file 1.** Role of the workshop participants and their relation to policy-making per country.

## Data Availability

The workshop discussions transcripts and SWOT analysis tables from the four individual workshops are available.

## Abbreviations

BDA: Big data analytics
EDR: EVOTION Data Repository
HA: Hearing aid
HL: Hearing loss
PH: Public health
PHPDM: Public Health Policy Decision-Making model
SWOT: Strengths, Weaknesses, Opportunities, Threats
