# Supplementary material for: Public health policy-making for hearing loss: stakeholders’ evaluation of a novel eHealth tool
Source: Health Res Policy Syst. 2020 Oct 29;18:125. doi: 10.1186/s12961-020-00637-2 (PMC7596974; doi:10.1186/s12961-020-00637-2)
Supplement: Supplementary file 1 — Additional file 1. Role of the workshop participants and their relation to policy-making per country. [file 12961_2020_637_MOESM1_ESM.docx]

# Additional file 1

Role of the workshop participants and their relation to policy-making per country.

| **Workshop country (number of participants)** | **Position/Institution** | **Expertise** | **Role/Involvement**  **in policy-making** | **Level**  **(regional/ national/**  **international)** |
| --- | --- | --- | --- | --- |
| **UNITED KINGDOM**  **N=5** | Independent consultant / Imperial College London | Audiology  eHealth | Advises policy makers at Digital Health, the NHS and provider services | National |
|  | University College London / Independent Consultant | Audiology | Advises on newborn hearing screening. | National/ international |
|  | NHS England London Region | Audiology | Policy-making in clinical leadership and Healthcare systems.  Interprets and delivers the NHS Long Term Plan.  Works with Chief Scientific Officer Senior leadership team and Medical Directorate team for London.  Collaborates with the National Chief Scientific Officer who has professional leadership for hearing. | National/ Regional |
|  | Public Health England | Public Health (PH) | Part of the team leading on thought leadership and implementation. | National |
|  | Health Education England / Independent Audiology Practice | Audiology | Member of the BSA Adult Rehab Interest Group – reviews policies such as recommended procedures or NICE guidelines | National |
| **CROATIA**  **N=7** | Osijek-Baranja County Administrative Department for Health and Social Welfare | Health policy | Development and implementation of health policy | Regional |
|  | Clinical Hospital Centre, Osijek | ENT | Consultant | Regional |
|  | University of Osijek | Biomedical statistics  Engineering | Academic | National |
|  | University of Osijek | Noise exposure | Academic | National |
|  | Croatian Pension Insurance Institute, Osijek | Pension insurance policy | Implementation of pension insurance policies | Regional |
|  | The SUVAG Polyclinic, Osijek | Noise  Audiology | Consultant | Regional |
|  | Association of the Deaf and Hard of Hearing of Osijek-Baranja County | PH | Planning of PH programs | Regional |
| **BULGARIA**  **N=6** | Ministry of Health, eHealth Directorate | eHealth | Advises on draft E-health legislation/regulations | National |
|  | Ministry of Labour and Social Policies | Public policy | Advises on draft PH policy legislation/regulations on HA reimbursement | National |
|  | Sofia Medical University, Occupational Medicine Department | Occupational Health | Advises PH policy experts | National |
|  | National Centre of Public Health and Analyses | Health Technology | Advises on PH policies | National |
|  | Association “National Patients’ Organisation” | PH | Advises PH policy experts;, e.g. in the Council of Ministers of Bulgaria  Supervises clinical trials of digital PH solutions | National |
|  | National Agency for People with Disabilities | Public Policy | Advises on draft legislation/regulations on disabilities (e.g. provision of HAs) | National |
| **POLAND**  **N=5** | Ministry of Health, Department of Public Health and Family | PH | Advises on PH policy legislation/regulation | National |
|  | National Health Fund, Health Care Services Department | Public Policy  HA financing | Advises on PH policy legislation/regulation | National |
|  | State Sanitary Inspection, Department of Social Communication and Health Promotion | Occupational/ Environmental Health | Advises on PH policy legislation/regulation | National |
|  | Central Institute for Labour Protection, Department of Vibroacoustic Hazards, Laboratory of Noise | Occupational/ Environmental Health | Advises on PH policy legislation/regulation | National |
|  | Polish Association of Audiologists | Audiology | Advises on hearing-impaired patients, health protection and promotion | National |
